# Interactions of resveratrol with other phenolics and activity against food‐borne pathogens

**DOI:** 10.1002/fsn3.1073

**Published:** 2019-05-29

**Authors:** Danijela Skroza, Vida Šimat, Sonja Smole Možina, Višnja Katalinić, Nataša Boban, Ivana Generalić Mekinić

**Affiliations:** ^1^ Department of Food Technology and Biotechnology, Faculty of Chemistry and Technology University of Split Split Croatia; ^2^ University Department of Marine Studies University of Split Split Croatia; ^3^ Biotechnical Faculty University of Ljubljana Ljubljana Slovenia; ^4^ Department of Clinical Epidemiology University Hospital Split and University of Split School of Medicine Split Croatia

**Keywords:** antimicrobial activity, pathogens, phenolics, phenolics interaction, resveratrol

## Abstract

The aim of this study was to investigate the antibacterial activity of individual phenolics and their binary mixtures with resveratrol against selected food‐borne pathogens. The antibacterial activity was quantified using the broth microdilution method by the determination of minimal inhibitory concentrations (MICs). Interactions between compounds in the binary phenolic mixtures were determined by calculating the fractional inhibitory concentration index (FICI). The influence of the number of OH groups in the phenols’ structure on their antibacterial activity was assessed by principal component analysis (PCA). The most effective compounds were flavone luteolin and flavonol rutin, while the weakest antimicrobial activity was observed for phenolic acid and flavan‐3‐ols (catechin and epicatechin). The synergistic effect (FICI ≤0.5) of equimolar mixture of resveratrol with kaempferol was confirmed against *Staphylococcus aureus*, *Bacillus cereus*, and *Escherichia coli*, while the mixture of rutin with resveratrol proved synergistic only against *S. aureus*. The increasing concentrations of resveratrol in the mixtures with kaempferol and rutin resulted in a loss of synergism which indicates that only selected phenolic mixtures, with optimal concentrations of their individual components, result in synergistic antibacterial activity. We did not find an association between total number of OH groups and antibacterial activity of either individual phenolics or their mixtures.

## 1 INTRODUCTION

The plant kingdom is a source of countless structurally diverse compounds, many of which have a strong antimicrobial activity. It is well known that some plant secondary metabolites occur as a response to a microbial infection, implying their antimicrobial activity on a wide range of microorganisms (Mostafa et al., 2018; Radulović, Blagojević, Stojanović‐Radić, & Stojanović, 2013; Rauha, 2001). Among them, phytoalexin resveratrol that is produced by different plants, such as grapevines and peanuts, is the most relevant and extensively studied. It has shown strong biological activity, such as in the case of the antibacterial and antioxidant effects of wine (Friedman, 2014; Radovanović, Jovančićević, Radovanović, Mihajilov‐Krstev, & Zvezdanović, 2012; Skroza, Generalić Mekinić, Svilović, Šimat, & Katalinić, 2015). However, different foods are rich in many other phenolic compounds with proven biological effects, including antimicrobial. By interacting with them, the biological activity of resveratrol could be altered (Friedman, 2014; Iacopini, Baldi, Storchi, & Sebastiani, 2008; Kurin, Mučaji, & Nagy, 2012; Skroza et al., 2015; Turan, Gulsen, Makris, & Kefalas, 2007; Del Valle et al., 2016).

The antibacterial activity of phenolic compounds is related to their structures and the type of microorganism (Kumar & Pandey, 2013; Shan, Cai, Brooks, & Corke, 2007). Due to large variability of the reactive groups in phenolics’ structure, their antimicrobial effects may be mediated by different mechanisms. In addition, different targets on the microorganism cells may be affected (Skandamis, Koutsoumanis, Fasseas, & Nychas, 2006; Xie, Yang, Tang, Chen, & Ren, 2015). Phenolics are capable of interacting with the cytoplasmic membrane, cell wall, nucleic acids, and/or energy transport, by altering or inhibiting their functions (Kumar & Pandey, 2013; Sanhueza et al., 2017; Xie et al., 2015). Furthermore, they have the ability to denature enzymes, or bind to vitamins, minerals, and carbohydrates making them inaccessible to microorganisms (Kumar & Pandey, 2013).

Many studies demonstrated the importance of the structure–activity relationship (SAR) in regard to the antibacterial activity of flavonoids (Friedman, 2014; Kumar & Pandey, 2013; Rauha, 2001; Sanhueza et al., 2017; Tripoli, Guardia, Giammanco, Majo, & Giammanco, 2007). For example, it has been shown that flavonoids without an OH group in the B ring (less polar molecules) have a stronger antibacterial activity than those without this structural feature (Friedman, 2014; Rauha, 2001). A large number of studies examining antimicrobial properties of different plant extracts assume that their overall effects are results of interactions of compounds contained in the extracts (Kim, Moon, & Lee, 2000; Mostafa et al., 2018; Park, Kim, Moon, & Lee, 1997; Radulović et al., 2013; Sanhueza et al., 2017; Tajkarimi, Ibrahim, & Cliver, 2010).

The aim of this study was to investigate the antibacterial activity of individual phenolic compounds from a group of phenolic acids, flavonols, flavones, and flavan‐3‐ols in relation to resveratrol against several food‐borne pathogens. In order to investigate possible synergistic, additive or antagonistic interactions of these compounds, they were used in combination with resveratrol as binary phenolic mixtures.

## 2 MATERIALS AND METHODS

### 2.1 Bacterial strains and growth conditions

Bacterial strains, *Bacillus cereus* WSBC 10530 (clinical isolate), *Staphylococcus aureus* ATCC 25923 (clinical isolate), *Salmonella* Infantis ŽM9 (poultry meat isolate), and *Escherichia coli* O157:H7 ŽMJ 129 (clinical isolate), were used for antibacterial testing. The cultivation medium for all used strains was Müeller Hinton Broth/Agar (MHB, MHA; Oxoid). The bacterial cultures were prepared by picking a colony from 24‐hr‐old MHA plates, and it was suspended in 4 ml MHB. The bacterial cultures were grown aerobically for 20 hr and at 37°C with continuous shaking at 100 rpm. For antibacterial activity assays, the suspensions were diluted in MHB medium to 10^5^–10^6^ CFU/ml.

### 2.2 Pure phenolic compounds and binary phenolic mixtures

The present study included commercially available phenolic compounds obtained from Sigma (Sigma–Aldrich GmbH): caffeic acid (95%, HPLC), protocatechuic acid, syringic acid (98%), rosmarinic acid (97%), *p*‐coumaric acid (98%), (+)‐catechin hydrate (98%, HPLC), quercetin, luteolin and *trans*‐resveratrol (99%, GC); and Fluka: *p*‐hydroxybenzoic acid (≥98%), vanillic acid (≥97%), gallic acid monohydrate (98%, HPLC), ferulic acid (≥98%), sinapic acid (≥97%), (−)‐epicatechin (≥90%, HPLC), kaempferol (≥96%, HPLC), and rutin trihydrate (≥95%, HPLC). The phenolic standards were dissolved in ethanol/water mixture (80:20, v:v) to the final concentration of 10 mM. In case of poorly soluble compounds (kaempferol, quercetin, and luteolin), the stock solutions were prepared in concentrations of 5 mM, while the concentration for rutin was 2.5 mM. Thus, prepared solutions were used for the preparation of binary mixtures with resveratrol.

### 2.3 Minimal inhibitory concentration (MIC) determination

For the broth microdilution test, 50 μl of each bacterial suspension was added to the wells of a sterile 96‐well microtitre plate (Nunc) already containing 50 μl of a twofold serially diluted pure phenolic compound or a binary phenolic mixture in MHB. The control wells were prepared with culture medium, bacterial suspension only, phenolic solution only, and ethanol in amounts corresponding to the highest quantity present. The contents of each well were mixed on a microplate shaker (Eppendorf) at 800 rpm for 1 min prior to incubation at 37°C. The MIC was the lowest concentration where no viability was observed after 24 hr. As an indicator of bacterial respiratory activity, the presence of color was checked visually after adding 10 μl/well of iodonitrotetrazolium chloride (INT, Sigma) dissolved in water (2 mg/ml) and incubated for 30 min in the dark (Klančnik, Piskernik, Jeršek, & Smole Možina, 2010). Bacterial growth was considered inhibited when the solution in the well remained clear. Positive controls (bacterial suspension with growth medium), negative controls (growth medium and pure phenolic compound or binary phenolic mixtures), and solvent controls (bacterial suspension with ethanol in amounts corresponding to the highest quantity present in the broth microdilution assay; 20%) were included in each experiment. All measurements were repeated in triplicate and mean values are given in tables.

### 2.4 Interaction and statistical analysis

Statistical analysis was performed using Statistica 8 (StatSoft Inc.) software package. Multivariate principal component analysis (PCA) was used to determine the influence of the number of OH groups in the phenols’ structure on their antibacterial activity. PCA is a multivariate mathematical approach which allows a visualization of similarities between observations and brings out patterns in analytical data sets.

The interaction between the compounds in relation to the antibacterial activity was determined by calculating the fractional inhibitory concentration index (FICI) (Balouiri, Sadiki, & Ibnsouda, 2016), which was calculated for each mixture using the following formula: FIC_A_ + FIC_B_ = FICI, where FIC_A_ = MIC of compound A in the phenolic mixture/MIC of compound A alone, and FIC_B_ = MIC of compound B in the phenolic mixture/MIC of compound B alone. A synergistic interaction was defined if the FICI value was 0.5 or less and an antagonistic interaction was if the FICI was over 4. The FICI values between 0.5 and 1 were interpreted as additive and between 1 and 4 as an indifferent interaction.

## 3 RESULTS AND DISCUSSION

### 3.1 Antibacterial activity of individual phenolic compounds

Analyses of antibacterial activity of individual phenolic compounds on selected gram‐positive (*B. cereus*, *S. aureus*) and gram‐negative (*E. coli*, *S.* Infantis) bacterial species, generally revealed that flavonoids and resveratrol were more effective than phenolic acids. Among them, the most effective, with the lowest MIC values, were flavone luteolin and flavonol rutin (Table 1). This is in line with observations by other authors who also demonstrated better activity of flavonoids relative to phenolic acids (Cueva et al., 2010; Radovanović et al., 2012; Sanhueza et al., 2017). One possible explanation for weaker antibacterial activity of phenolic acids is less polarity of these compounds, which diffuse more slowly into the culture medium (Klančnik et al., 2010; Moreno, Scheyer, Romano, & Vojnov, 2006). Interestingly, flavan‐3‐ols, catechin, and epicatechin proved least effective among the tested flavonoids. This is in agreement with the results of Cueva et al. (2010), Gomes et al. (2018), Sanhueza et al., (2017) and Shan (2008) who also did not observe noticeable antibacterial activity of catechin (MIC >3,445–34,450 µM) against same pathogens. Stilbene resveratrol showed conspicuous activity against the tested gram‐positive bacterial species, and somewhat weaker activity against the gram‐negative species. A similar trend in the antibacterial activity of resveratrol regarding gram‐positive and gram‐negative staining was observed by Taguri, Tanaka, and Koundo (2006) and Shan (2008). Taguri et al. (2006) reported the MIC values for resveratrol against gram‐positive bacteria (*B. cereus* and *S. aureus*) of more than 9,000, and 14,000 μM for gram‐negative bacteria (*E. coli*). In the study by Shan (2008), the MICs were 2,740 µM against *E. coli* and 1,370 μM for *Salmonella* and gram‐positive *B. cereus* and *S. aureus*.

**Table 1:** The antibacterial activity of selected phenolic compounds, expressed as the MIC value in µM

Sample	MIC (µM)			
	Gram+		Gram−	
	*S. aureus*	*B. cereus*	*E. coli*	*S.* Infantis
Phenolic acids				
*p*‐hydroxybenzoic	2,500.0	1,250.0	1,250.0	1,250.0
Protocatechuic	2,500.0	1,250.0	2,500.0	2,500.0
Vanillic	2,500.0	625.0	625.00	1,250.0
Syringic	2,500.0	1,250.0	1,250.0	1,250.0
Gallic	1,250.0	1,250.0	1,250.0	1,250.0
*p*‐ coumaric	1,250.0	625.0	625.0	1,250.0
Caffeic	1,250.0	1,250.0	625.0	625.0
Ferulic	2,500.0	1,250.0	2,500.0	2,500.0
Sinapic	1,250.0	1,250.0	1,250.0	625.0
Rosmarinic	1,250.0	1,250.0	1,250.0	1,250.0
Flavan‐3‐ols				
Catechin	1,250.0	1,250.0	625.0	625.0
Epicatechin	2,500.0	1,250.0	2,500.0	2,500.0
Flavonols				
Kaempferol	312.5	312.5	1,250.0	1,250.0
Quercetin	312.5	625.0	312.5	312.5
Rutin	312.5	156.3	156.3	156.3
Flavon				
Luteolin	156.3	312.5	312.5	312.5
Stilbene				
Resveratrol	312.5	312.5	625.0	625.0

Concerning the structure–antibacterial activity relationship, although containing the highest number of free OH groups in their structure, catechin and epicatechin showed a weak effect which was comparable to that of phenolic acids. Moreover, despite the same number of OH groups, catechin was more active than its epimer, particularly against gram‐negative species. On the other hand, quercetin with two OH groups was more effective against gram‐negative bacteria than kaempferol with one free OH group in ring B. The glycosylation of ring C at position 3 in quercetin, forms rutin which resulted in enhanced activity toward both, *B. cereus* and gram‐negative species (*E. coli*, *S.* Infantis) (Table 1). A comparison of the selected flavonoids with the catechol group in B ring (catechin, quercetin, luteolin, rutin) suggest that the presence of free OH groups in that ring does not play a significant role in their antibacterial activity. Instead, the dominant factor could be a difference in the structure of ring C, like the presence or absence of OH and/or keto groups (Kumar & Pandey, 2013; Taguri et al., 2006). Except structural differences, all these compounds possess different mechanism of action on microbial cell. It has been described that catechin induced cytoplasmic damage, that quercetin can cause an increase in the permeability of the cytoplasmic membrane, and that lueolin affected the cytoplasmic membrane stability and inhibited enzymes (Sanhueza et al., 2017).

**Figure 1:**
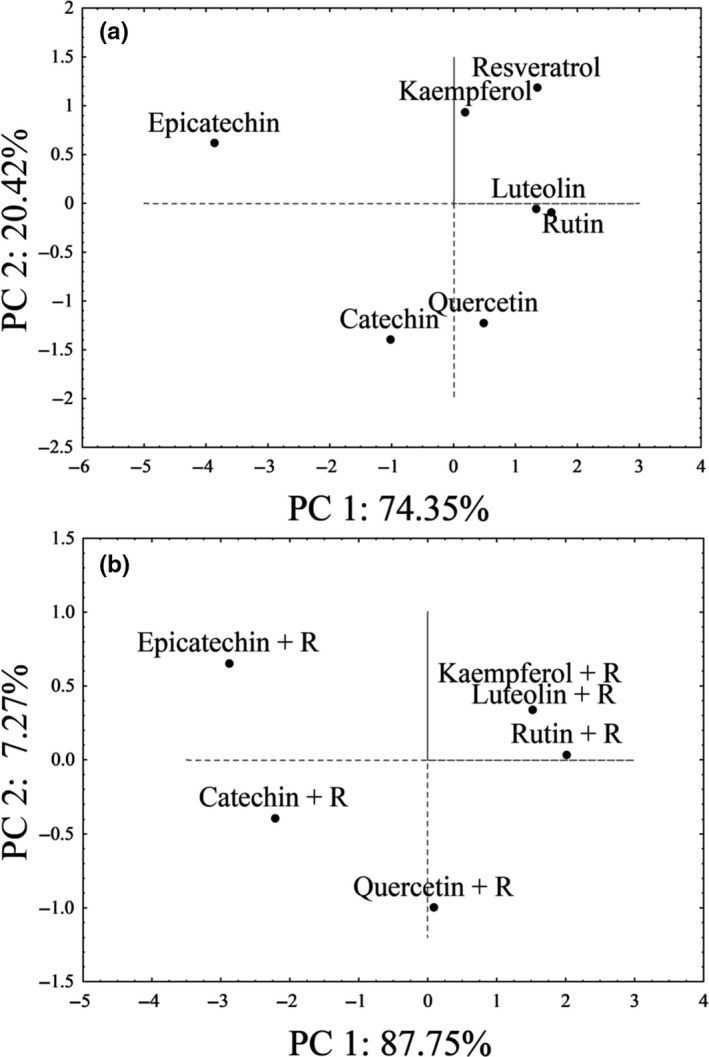
(a) Principal component analyses (PCA) for antibacterial activity (PC1) and number of free OH groups for selected individual phenolic compounds (PC2). (b) PCA for antibacterial activity (PC1) and number of free OH groups (PC2) for selected phenolic mixtures with resveratrol (R)

The results from the antibacterial activities of the tested phenolic compounds against gram‐positive and gram‐negative bacterial species were analyzed by PCA. Figure 1a shows the position of polyphenols in the multivariate space defined by PC1 and PC2 which describes 94.77% of the variability between the data. Considering the influence of PC1 (antibacterial activity), it is obvious that the most effective phenolics were grouped on the right side, while catechin and epicatechin as the weakest antibacterial agents were positioned on the left part of the graph. Despite the specific location of the cases in the multivariate space, the impact of the OH group presence (PC2) on the antibacterial activity of phenolics was not confirmed. For example, resveratrol and kaempferol, regardless of the difference in the number of OH groups, were grouped in the same quadrant based on their antibacterial activity. Further, the compounds that showed the best activity against the tested bacterial species (luteolin and rutin) have four OH groups while catechin, epicatechin, and quercetin with five OH groups showed lower antibacterial activity. Consequently, we could not confirm that the catechol structure feature in ring B enhances the antibacterial activity of the compound. This is in line with the findings of Kumar and Pandey (2013) and Taguri et al. (2006) who also could not establish a clear relationship between the total number of OH groups and antibacterial activity.

### 3.2 Antibacterial activity of binary phenolic mixtures

Despite numerous studies examining the antibacterial effects of combined natural compounds such as essential oils (Palaniappan & Holley, 2010; Patrone, Campana, Vittoria, & Baffone, 2010; Periago & Moezelaar, 2001; Rivera Calo, Crandall, O'Bryan, & Ricke, 2014) or phenols and organic acids (Del Valle et al., 2016; Vasconcelos de Oliveira, Montenegro Stamford, Gomes Neto, & Leite de Souza, 2010), information on the interaction of individual phenolic compounds with resveratrol are rather scarce (Chan, 2002; Del Valle et al., 2016). In this study, the antibacterial activity of selected phenolic compounds with resveratrol was tested by the microdilution method, and their interaction as binary phenolic mixtures was described by the fractional inhibitory concentration index (FICI) (Mundy, Pendry, & Rahman, 2016; Palaniappan & Holley, 2010; Petersen, Labthavikul, Jones, & Bradford, 2006). The data presented in Table 2 indicate that mixing phenolic acids with resveratrol at an equimolar ratio generally improved their antibacterial activity. The FICI index above 0.5 and lower than 4 indicates that the interactions of phenolic acids with resveratrol (Table 2) are additive or indifferent in nature. Regarding equimolar mixtures of flavonoids with resveratrol, the best results were obtained with flavonols kaempferol and rutin. The synergistic effect (FICI ≤0.5) of resveratrol with kaempferol mixture was confirmed against *S. aureus*, *B. cereus*, and *E. coli*, while for *S.* Infantis the effect was additive. The synergistic effect of rutin with resveratrol was demonstrated only against *S. aureus* (Table 2).

**Table 2:** The antibacterial activity of mixtures of resveratrol with selected phenolic compounds at a molar ratio 1:1 (expressed as the MIC value in µM)

Resveratrol +	Gram+				Gram−			
	*S. aureus*		*B. cereus*		*E. coli*		*S.* Infantis	
	MIC	FICI	MIC	FICI	MIC	FICI	MIC	FICI
Phenolic acids								
*p*‐hydroxybenzoic	625.0	1.13	625.0	1.25	625.0	0.75	625.0	0.75
Protocatechuic	625.0	1.13	625.0	1.25	625.0	0.63	1,250.0	1.25
Vanillic	625.0	1.13	625.0	1.5	625.0	1.00	1,250.0	1.50
Syringic	625.0	1.13	625.0	1.25	625.0	0.75	625.0	0.75
Gallic	625.0	1.25	625.0	1.25	625.0	0.75	625.0	0.75
*p*‐ coumaric	625.0	1.25	625.0	1.50	625.0	1.00	1,250.0	1.50
Caffeic	625.0	1.25	625.0	1.25	625.0	1.00	625.0	1.00
Ferulic	625.0	1.13	625.0	1.25	1,250.0	1.25	1,250.0	1.25
Sinapic	625.0	1.25	625.0	1.25	625.0	0.75	1,250.0	2.00
Rosmarinic	625.0	1.25	625.0	1.25	625.0	0.75	1,250.0	1.50
Flavan‐3‐ol								
Catechin	625.0	1.25	625.0	1.25	625.0	1.00	1,250.0	2.00
Epicatechin	625.0	1.13	625.0	1.25	1,250.0	1.25	1,250.0	1.25
Flavonols								
Kaempferol	156.3	**0.50**	156.3	**0.50**	312.5	**0.38**	625.0	0.75
Quercetin	312.5	1.00	312.5	0.75	312.5	0.75	625.0	1.50
Rutin	156.3	**0.50**	156.3	0.75	156.3	0.63	312.5	1.25
Flavon								
Luteolin	156.3	0.75	312.5	1.00	312.5	0.75	312.5	0.75

Note

The interactions between the compounds in the mixtures in relation to the antibacterial activity are expressed as Fractional Inhibitory Concentration Index (FICI) values. FICI ≤0.5 indicates a synergistic interaction, FICI = 0.5–1.0 additive, FICI = 1.0–4.0 indifferent interaction and FICI >4.0 indicates antagonism among the tested phenolic compound.

The bold FICI values indicate synergistic effect of the phenolic mixtures.

**Figure 2:**
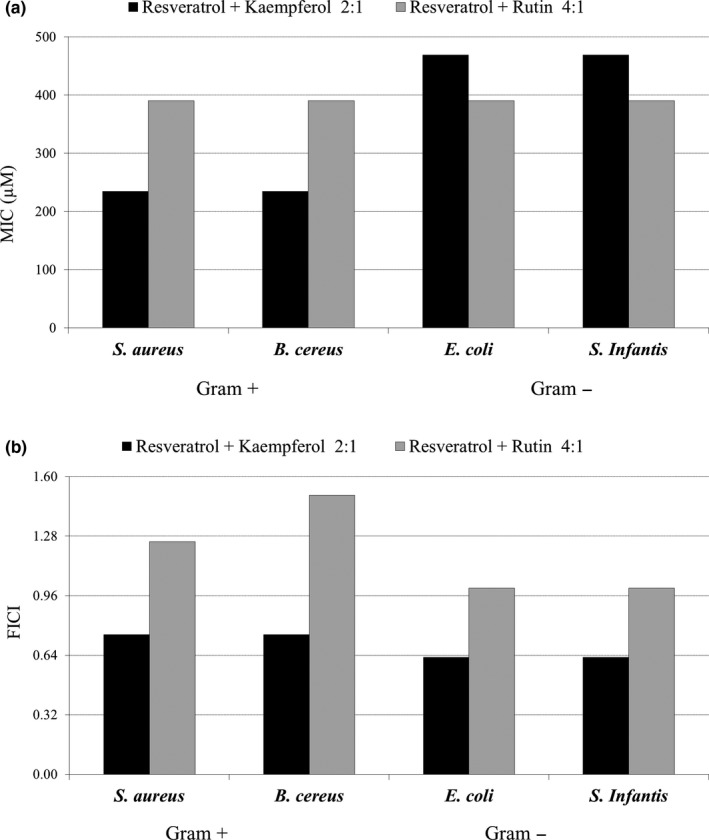
(a) The effects of increasing doses of resveratrol in the binary phenolic mixtures on their antibacterial activity. The results are shown for mixtures of resveratrol with kaempferol and with rutin at 2:1 and 4:1 molar ratios, respectively (expressed as MIC values in µM); (b) The interaction between the compounds in the same binary phenolic mixtures described by the fractional inhibitory concentration index (FICI)

In order to examine the effects of increasing doses of resveratrol in binary phenolic mixtures, the antibacterial activity of mixtures of resveratrol with kaempferol and resveratrol with rutin were tested at 2:1 and 4:1 molar ratios, respectively. The MIC values and FICI index for these mixtures are shown in Figure 2. The mixture of resveratrol with kaempferol at the 2:1 molar ratio resulted in higher MIC and FICI values for all tested microorganisms, compared to the mixture of the same compounds at a 1:1 molar ratio (Figure 2, Table 2.). The resveratrol and rutin mixture, where the resveratrol fraction was increased fourfold (4:1 molar ratio), also resulted in a loss of synergism and diminished antibacterial efficacy. Taken together, these findings indicate that only selected phenolic mixtures, and at optimal concentrations of individual components, result in synergistic antibacterial activity.

The results from the antibacterial activities of the tested phenolic mixtures were also analyzed by PCA. Figure 2b shows the position of polyphenolic mixtures with resveratrol in the multivariate space, specifically with mixtures of resveratrol with kaempferol, luteolin, and rutin grouped in the same quadrant. These mixtures containing seven OH groups showed better antimicrobial activity than the remaining three mixtures with eight OH groups. This again is indicative that antibacterial activity cannot simply be estimated by the number of OH groups, it is necessary to take into account other factors, such as position of OH groups, solubility, polarity, medium pH, and bacterial properties.

## CONFLICT OF INTEREST

The authors declare that they have no conflict of interest.

## ETHICAL STATEMENT

The study did not involve any human or animal testing.
